# Palliative Care Spiritual Assessment and Goals-of-Care Discussions in the Neurocritical Care Unit: Collaborating with Chaplains

**DOI:** 10.1007/s12028-024-02190-0

**Published:** 2025-01-23

**Authors:** Allison Kestenbaum, Danielle Gilchrist, Brian C. Dunlop

**Affiliations:** 1 University of California, San Diego Health, San Diego, CA USA; 2 Temple University Health System, Philadelphia, PA USA; 3 Pennsylvania Hospital, University of Pennsylvania Health System, Philadelphia, PA USA

**Keywords:** Palliative, End of life, Spiritual care

## Abstract

**Introduction:**

Neuropalliative care is an emerging subspecialty of palliative care designed to address the unique supportive care needs of patients with serious neurological illness, including those receiving neurocritical care in intensive care units. Spiritual care is a vital component in the provision of holistic and humanized care to these patients. A chaplain who is specially trained and credentialed in care for those with serious illness is the health care professional responsible for making spiritual assessments and contributes to the plan of care, facilitating decision making, and guiding other clinicians in the provision of generalist spiritual care.

**Methods:**

This article illustrates the role of chaplains in supporting neurocritical care patients and highlights two fundamental aspects of spiritual care: (1) spiritual screening/assessment and (2) assistance with goals-of-care conversations.

**Results:**

These cases clarify the role of professionally trained and credentialed chaplains with experience in both neurocritical and palliative care and the value added to the interprofessional team.

## Introduction

Neuropalliative care is an emerging subspecialty of palliative care designed to address the unique supportive care needs of patients with serious neurological illness, including those receiving neurocritical care in intensive care units [1, 2]. The recent growth of neuropalliative care reflects recognition that these patients and their entire ecosystem of caregivers, families, friends, and clinicians benefit from the holistic approach that palliative care provides [3]. This approach to care includes attending to the spiritual and religious needs of family members of patients who present with devastating neurologic injuries that render them unable to communicate their needs or participate in treatment decision-making and goals-of-care conversations [4]. These patients and their caregivers frequently experience spiritual distress as well as existential suffering as they navigate a sudden threat to their life or quality of life, prognostic uncertainty, and sudden unexpected changes to image, independence, mobility, and morbidity.

Spiritual care is a vital component in the provision of holistic and humanized care to patients who are critically ill. The National Consensus Project for Quality Palliative Care recognizes spiritual, religious, and existential aspects of care as one of the eight core domains of quality palliative care. All clinicians caring for patients who are seriously ill can provide generalist spiritual care as a fundamental tool in the provision of comprehensive care to neurologically injured patients [5, 6]. However, a chaplain who is specially trained in providing care to those with serious illness is the health care professional expert responsible for making spiritual assessments, contributing to the plan of care, and facilitating decision-making. This article illustrates the role of chaplains in supporting neurologically ill or injured patients in intensive care unit settings receiving palliative care and highlights two fundamental aspects of spiritual care: (1) spiritual screening/assessment and (2) assistance with goals-of-care conversations. This article also provides guidance to interprofessional neurocritical care clinicians about how they can identify and address spiritual needs and when chaplain specialist support is needed. These cases clarify the role of professionally trained and credentialed chaplains with experience in both neurocritical and palliative care and the value added to the interprofessional team.

## Spiritual Screening and Assessment

All health care professionals should be able to conduct a brief spiritual screening to determine how a patient’s spiritual and/or religious beliefs may impact their plan of care. These are not time intensive and are designed to be asked at any point in the hospitalization. Spiritual screening entails one question or a brief decision tree that any clinician (not just the chaplain) should use to identify aspects of the patient’s or family’s spiritual and religious background that may impact care and their current state of well-being. There are several one-sentence tools that have been developed and studied in palliative care contexts, and the tools in Table 1 are recommended by the authors [7, 8]. We recommend these tools in part because they contain a threshold for referral to a chaplain. An important competency for clinicians is to identify when these screenings indicate referral to a professional chaplain, and tools that partially automate this process can be helpful in busy neurocritical care units. Skillful triaging is required because although chaplains demonstrate dozens of competencies that support patient care and team functioning, the level of staffing and integration into organizational and team protocol in palliative and neurocritical care varies greatly across institutions [9–11].

**Table 1:** Spiritual screening, spiritual history, spiritual assessment

	Definition	Who conducts	Evidence-based examples
Spiritual screening	A single question or brief decision tree that screens for spiritual (including religious) pain, struggle, and resources	Any clinician	1. “On a scale of 0–10, please rate your level of spiritual pain. Spiritual pain is pain deep in your soul/being that is not physical.” [7] (3 + triggers chaplain assessment) 2. “Are you at peace?” [19] (“yes” triggers chaplain assessment) 3. Rush Protocol [20] 4. “Do you have any spiritual or religious practices that are important to you?”[8] (“yes” triggers chaplain assessment)
Spiritual history	Series of open-ended questions (often an acronym) about spiritual and religious beliefs and practices	Any clinician	FICA [13] HOPE [21]
Spiritual assessment	Most often triggered by spiritual screening/history and referral to chaplain; not a question but rather a dynamic model for diagnosing and addressing specific forms of spiritual distress (e.g., broken relationships, lack of peace, loss of meaning/purpose) through corresponding interventions, resulting in observable outcomes	Chaplain	Two models that have been used/studied in palliative care: 1. Spiritual AIM [14]: Spiritual needs are: •Meaning/direction •Reconciliation/love •Self-worth community Chaplain responds with core professional strategies, such as counseling, appropriate rituals, spiritual practices/community informed by spiritual AIM that correspond with specific spiritual need and provide guidance, affirmation, and/or accountability 2. PC-7 [22]: Spiritual needs are: •Need for meaning in the face of suffering •Need for integrity, a legacy, generativity •Concerns about relationships: family and/or significant others •Concern/fear about death or dying •Issues related to making decisions about treatment •R/S struggle Chaplain responds with core professional strategies based on assessment made; no corresponding interventions proscribed by model

AIM, Spiritual Assessment and Intervention Model

Many medical providers are aware that the spiritual dimension of care and patients’ views and values impact healing and medical decision-making. They may even recognize that a majority of seriously ill patients would like their medical providers to know about their spiritual and religious beliefs and practices [8, 12]. Medical providers who have adequate time and rapport with a patient and their family can obtain more in-depth spiritual information through a spiritual history (Table 1). Spiritual histories may reveal more about who the person was before their illness and cultivate empathy and compassion in the caregiver toward the patient as a whole person. Once this information is revealed, interprofessional clinicians can engage with this information as they would any other core markers of the patient’s identity. Spiritual histories also allow clinicians to proactively elicit information about how spiritual and religious beliefs and values may impact attitudes and preferences about routine aspects of neurocritical care, such as patient/family well-being, pain management, medical decision-making, palliation, and end-of-life care [13]. To summarize, spiritual screening and spiritual histories are the tools that reveal spiritual needs and resources, can help the primary team manage those needs, and may trigger a referral to a chaplain to conduct a full spiritual assessment.

Spiritual assessment is a separate approach conducted by chaplains, who are the spiritual care specialists on the team (Table 1). Spiritual assessment is one instrument in the professional chaplain’s tool kit wherein the chaplain assesses a primary area of spiritual suffering that needs attention. They do this by observing patient behavior, listening to what they say (if they are able to communicate), and gathering information about the person from the care team and loved ones.

This is a dynamic process that entails diagnosing the sources and severity of spiritual distress [14]. Chaplains assess a core spiritual need for the patient and develop a plan of care accordingly. A chaplain should be able to concisely describe this primary spiritual need, the specific actions and interventions to address it, and the outcomes that the medical team can expect to observe. In addition, all of this is documented in the electronic health record. Although there are several tools that are referred to as spiritual assessment models, those models that have been studied in the palliative clinical environment and offer suggested interventions and outcomes, along with guidance for making an assessment, are the preferred frameworks for use with neurocritical care patients [14, 15]. Spirituality and religion are expressions of culture, so palliative care chaplains are trained to identify of how patients cultural values, practices, and social location impacts how patients and their loved ones approach and make decisions about their medical treatment [16]. In the neurocritical care setting, chaplains who have completed thorough spiritual assessments are advocates for culturally humble care, including appropriate rituals and accommodations.

The case studies to follow demonstrate scenarios in which spiritual and religious needs and practices are integrated into the care plan and the chaplain interacts with neurocritical care staff:

- Case 1: A scenario in which a palliative care chaplain is available and spiritual needs (which are not religiously based) are clear and integrated into the care plan with relative ease
- Case 2: A scenario in which misunderstandings between the care team and the patient/family related to spiritual/religious aspects of care were brought to light and addressed with chaplain support with more limited availability

## Case 1: Spiritual Screening and Assessment in Action

Mrs. A is a 50-year-old African American woman hospitalized following a subarachnoid hemorrhage. She underwent emergency surgery and was treated in a neurocritical care unit. The patient has been visited by friends, and her husband has been at her bedside frequently. Her bedside nurse conducted a spiritual screening using the Rush Protocol (Table 1). The social worker in the neurointensive care unit conducted the FICA spiritual history with the patient’s husband. He noted that the patient considers herself spiritual but not religious and draws on meditation and prayer practices for spiritual support. The husband requested guided meditations as well as prayers for peace and healing. This information triggered a referral to the palliative care chaplain.

A palliative care chaplain visited Mrs. A when she was alert and conducted a Spiritual Assessment and Intervention Model (AIM) spiritual assessment (Table 1). The assessment included conversation and questions to determine the role of the patient’s spiritual beliefs in coping with and making decisions related to their illness, including rituals and spiritual practices. Spiritual counseling in acute settings focuses on the primary concern of the patient in that moment, as opposed to an extensive history-taking [17]. Visits in high acuity patient care areas of the hospital are likely to be interrupted at any time by other visiting members of the care team, family members, or the patient’s own exhaustion and need for rest. As spiritual distress can be expressed in many ways (e.g., not just religious), the chaplain asks a general, open-ended question about how things are going for the patient today. Mrs. A: “Although I don’t belong to a church or anything, my faith has always been very strong and has been strong throughout all of this. But I’ve definitely been trying to understand the meaning of this and why it happened.”

The chaplain recognizes that questions about searching for answers about purpose in suffering is a sign of a spiritual need for meaning and direction in the spiritual AIM assessment framework*.* The chaplain restates the patient’s feelings to help her clarify, without judgment, interpretation or try to solve the issue.

Chaplain: “So you are finding it difficult to connect with meaning and purpose in your life since this happened?”.

Mrs. A: “Yes. My faith is still very strong. And I am certain that I will find peace again. But I just can’t understand why this happened!? After what my sister went through, I couldn’t believe this! All in one year. She died suddenly. We miss her so much”.

The chaplain acknowledges the grief for the patient and family using the technique of normalizing the sadness and feelings of uncertainty about the purpose of all of this loss. While this may seem basic, grieving individuals may worry that their reactions to loss are abnormal, so they benefit from the chaplain normalizing and empathizing with their emotional and spiritual pain. As the chaplain has assessed the patient’s need for meaning and direction, the chaplain intervenes by reflecting back the patient’s questions and acting as a guide as they explore the ambiguity and lack of clarity in their situation.

Chaplain: “Looking at your sister’s death, and this illness, the meaning is not clear to you right now.”

When patients who seek meaning are grappling with spiritual distress, they benefit from caregivers who can accompany them while they seek direction and reconnect with values that have provided them with clarity in the past. A trained chaplain has the time and emotional self-awareness to fill this role for patients. To understand the chaplain’s role, one metaphor is that of walking on a path alongside the patient, not leading them, but metaphorically “shining a light” on the path so the patient can continue to express themselves.

The chaplain guides the patient to articulating her resources for coping and what is most important to her, including her roles in her family, in her religious community, and at work. She expresses gratitude, one of the spiritual AIM outcomes for patients with a primary need for meaning and direction. Gratitude may seem like a surprising outcome for a critically ill person, but it demonstrates an important source of coping and emotional/spiritual integration. Gratitude can exist in tandem with fear, grief, anger, and uncertainty.

Mrs. A: “I am very grateful though. I am somehow making it through this. I have so many blessings. I’m alive, and I have all of these people. Chaplain, will you pray with me?”.

When chaplains pray with patients, they draw on the customs, sacred text, and prayer structure from their cultural and religious backgrounds. In addition, chaplains offer interfaith “extemporaneous” or “custom-made” prayers or blessings to speak to the specific situation and spiritual needs of the moment.

Chaplain: “Mrs. A, I am amazed that you can find gratitude and reconnect with your purpose with all you are going through. Let us pray. We know that even when we find it hard to connect, Mrs. A’s hope and gratitude are strong. We pray that her physical recovery is sustained. We pray for comfort for her and her family as they grieve so many losses. Please allow her to continue to be surrounded by people she loves so that she can gain greater clarity about her purpose as she heals.”

Mr. A: “I didn’t know that she was having so much doubt. So, thank you. Is this very common with people who have gone through something like this…I mean…do you hear stuff like this a lot with patients who have gone through this?”.

The chaplain validates that this is in fact common and a normal part of spiritual healing in the face of a sudden serious illness. As the patient rests, the chaplain and Mr. A continue to discuss how Mrs. A had helped him to understand and express his own faith and that it was challenging to see her express doubt. The chaplain concludes by asking if they would like a follow-up visit and lets them know approximately when they can expect another visit and reminds them they can ask the nurse to page the on-call chaplain if needed. The chaplain documents the encounter in the electronic health record and verbally summarizes the patient’s needs and resources for the bedside nurse and social worker, who tell the chaplain they are helped by knowing more about the patient’s struggles and sources of strength.

## Case 2: Spiritual Care and Goals-of-Care Discussions

Mr. B is a 73-year-old man of Christian faith originally from Central America with minimal medical history. Mr. B was found unresponsive in a parked vehicle. Imaging showed a large intraparenchymal hemorrhage, and he was transferred to the comprehensive stroke center. The majority of Mr. and Mrs. B’s family was residing in Guatemala, and Mrs. B remained the legal surrogate, received updates, signed consents, and voiced the family’s decisions throughout the hospitalization. Mrs. B brought their faith front and center during the first interaction with the neurology intensivist physician. The physician asked Mrs. B medically related questions, and the majority of her answers referenced “God’s will.” Mrs. B expressed that the questions from the doctor were distracting her from remaining hopeful and faithful. The physicians expressed feeling at an impasse because they could not receive sufficient information from her to guide the care. The medical team scheduled a family meeting to discuss goals of care. They invited the palliative care chaplain, who was not able to attend due to short staffing and another pressing issue in the health system but provided guidance and coaching to the team about how to address spiritual and religious beliefs. The chaplain helped the medical team to understand that expressing faith is a source of empowerment and a cultural requirement, and a necessary prerequisite before they could allow themselves to discuss the actual condition of the patient. The chaplain also explained to the medical team that prior to the meeting, they could speak with the family’s pastor, who was advising them but was not fully informed about the medical realities of the situation. The chaplain coached the medical team about ways to address the belief that everything is “in God’s hands.” For example, if the family responded this way to discussions about code status, the medical providers could be curious about what this means to them.

Chaplain: “You may say something to them like ‘Some people say in God’s hands means if the patient’s heart stops and he dies, that’s God. They do not want Cardiopulmonary resuscitation (CPR) or attempts to have their heart restarted. Other people say medical providers are instruments of God, and they would like CPR and attempts at their heart being restarted. Please help us understand what you believe.”.

The medical team came to understand that the patient’s family was afraid and overwhelmed by the unknown. The chaplain indicated that although they could not come to the family meeting, they would meet with the patient’s wife prior to the goals-of-care meeting. The chaplain conducted a PC:7 spiritual assessment and identified the wife’s need for integrity and fear of death/dying (Table 1). Mrs. B indicated that she has a secure relationship with God. She needed to make decisions in integrity with this. Her religion was a primary source of strength that she needed to be able to discuss openly with all members of the medical team [18]. She stated that she believed in an afterlife and was certain that if God decided it was time for her husband to die, that he would be going to heaven, which brought her comfort. The chaplain prayed with Mrs. and Mr. B. They also discussed sacred Christian text that centered around drawing on faith and obtaining wisdom in the face of uncertainty.

The chaplain summarized these interventions in documentation in the medical record and spoke with the neurological intensivist with this information prior to the family meeting, serving as an additional conduit of communication. The neurocritical care team began the meeting with acknowledgment of the importance of the family’s faith, their need to stay true to this, and their fears and grief about the potential death of Mr. B. They invited Mrs. B to retell her story of this ordeal, reflecting back her feelings and values. Feeling supported by the chaplain and more seen and heard by the medical team, Mrs. B divulged that she felt she needed more time to see if Mr. B would recover to a quality of life that was acceptable to him. However, she admitted that if her husband were to live in a state in which he would be unable to interact, take care of himself, or feed himself, that would amount to a poor, unacceptable quality of life for Mr. B. Mr. B valued his independence and ability to care and provide for his family. Mrs. B’s biggest fear was that her husband “would be alive but have no life.” She chose to continue treatment and readdress the patient’s recovery, quality of life, and direction of care on a weekly basis. The chaplain supported the family during this uncertain time through making and collecting stories of Mrs. B’s favorite memories, his legacy as a faithful and loving person, significant experiences, and family bonds. The chaplain also provided emotional and spiritual support to the medical team, especially bedside nurses, who expressed feelings of moral distress in continuing Mr. B’s treatment. After two weeks of life-prolonging treatment, the patient showed no signs of recovery. The medical team saw Mr. B’s chances of being able to “walk, talk, and eat again” diminishing. Mrs. B’s fear of him “being alive but with no life” increased, so the family ultimately decided to transition the patient to comfort-directed care. Although they grieved openly, they expressed comfort in knowing that the decisions they made were concordant with the patient and family’s faith and values.

## Conclusion

Palliative care chaplains add a unique contribution to the holistic care of neurocritical care patients. Clinicians of all disciplines can conduct spiritual screening and history that trigger full spiritual assessment conducted by chaplains. Such assessments contribute to a plan of care and goals-of-care discussions that more fully consider the patient’s values, preferences, and key sources of spiritual and emotional support in making health care decisions.
